# End-stage kidney disease: a never healing wound leading to another never healing wound, renal cancer

**DOI:** 10.1007/s40620-023-01694-w

**Published:** 2023-07-13

**Authors:** Janos Docs, Gyula Kovacs, Lehel Peterfi

**Affiliations:** 1 Department of Urology, Medical School, University of Pecs, 7621 Pecs, Hungary; 2 Medical Faculty, Ruprecht-Karls-University, 69120 Heidelberg, Germany

**Keywords:** End-stage kidney disease, Cytokines, Fibroblast, Chronic inflammation, Tissue remodelling, Carcinogenesis

## Abstract

**Background:**

End-stage kidney disease and acquired cystic kidney disease are the final stages of chronic kidney disease, leading to loss of kidney function and frequent development of tumours. It has been suggested that an inflammatory microenvironment may be responsible for the progressive kidney remodelling and cancer development.

**Methods:**

Our aim was to analyse gene expression suggested to be involved in the remodelling of kidneys in end-stage kidney disease, and in the development of preneoplastic lesions and tumours. Immunohistochemistry was employed to assess the cellular localisation of different genes involved in these pathways on representative tissue sections.

**Results:**

Cellular (αSMA positive naïve activated fibroblasts, endothelial cells, macrophages) and non-cellular components (cytokines IL6, TGFβ, IL1β, CSF2, fibronectin, laminin, and matrix modifier proteases MMP9 and MMP12) of the inflammatory microenvironment were expressed in the kidneys of patients with end-stage kidney disease. IL6 and FN1 expressing naïve activated fibroblasts and recruited inflammatory cells were the most abundant cellular components of the inflammatory microenvironment.

**Conclusion:**

The progressive inflammatory and fibrotic processes in end-stage kidney disease have features recalling those of  a never healing wound and may explain the frequent development of kidney cancer.

**Graphical abstract:**

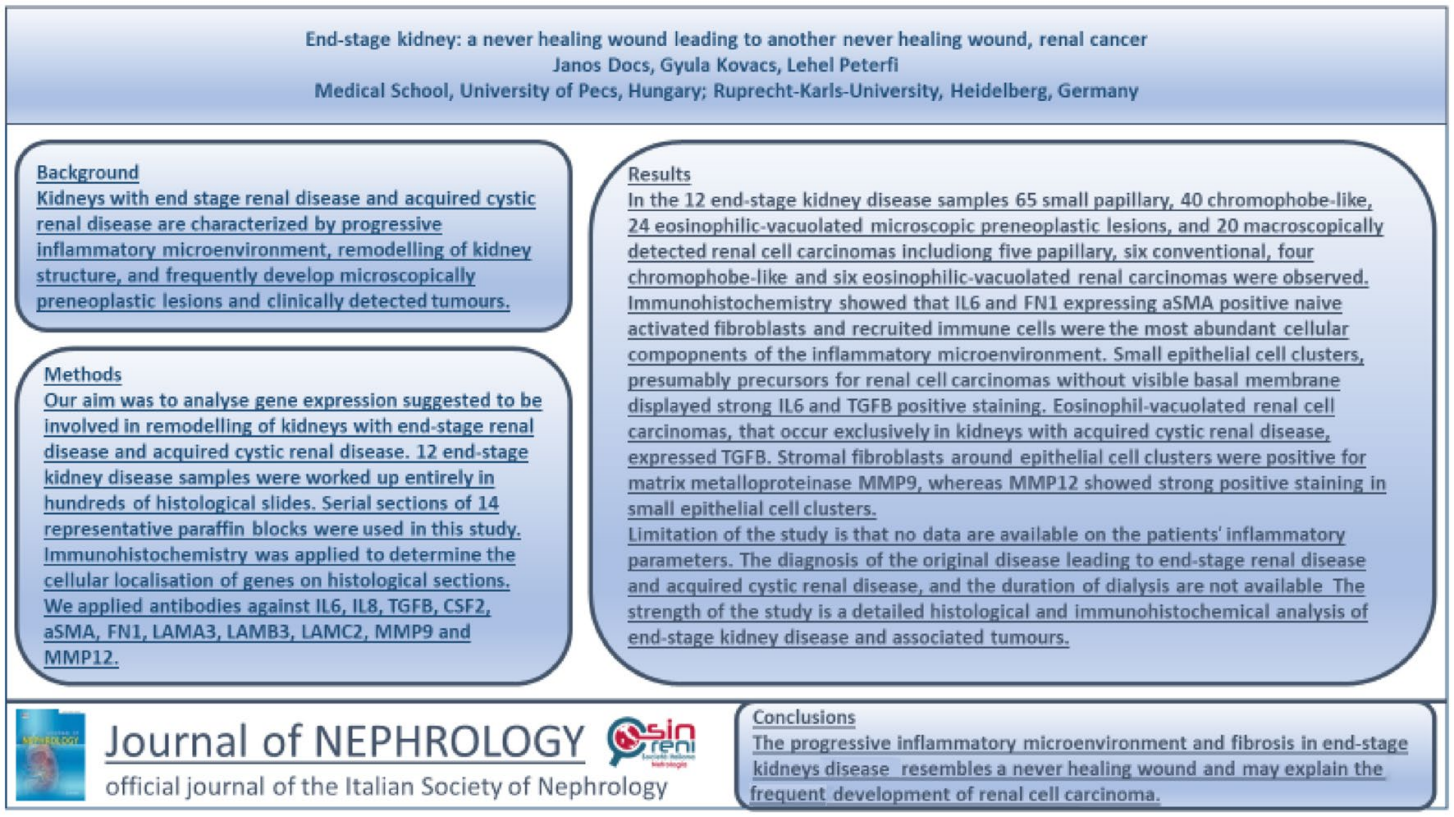

## Introduction

End stage kidney disease (ESKD) is the final stage of chronic kidney disease caused by different causes, including diabetes, hypertension, glomerulonephritis, drug- and toxin-induced tubulointerstitial nephritis, among others [1]. End-stage kidney disease is characterised by chronic interstitial inflammation and considerable stromal fibrosis replacing the normal kidney parenchyma [2]. After long-term intermittent maintenance haemodialysis, kidneys may undergo diffuse cystic changes leading to acquired cystic renal disease (ACKD) [3, 4]. Renal cell carcinoma (RCC) develops more frequently in patients with ESKD/ACKD than in the general population. Several ESKD/ACKD-associated tumours display unusual histology and genetic alterations that have never been observed before [5, 6]. An alternative pathway of tumourigenesis has been proposed to explain the high number of tumours in ESKD/ACKD [7]. It has been suggested that the inflammatory microenvironment of ESKD/ACKD may be responsible for the frequent development of a unique type of renal cell cancer. A global gene expression analysis of the kidneys of patients with ESKD/ACKD revealed a characteristic expression pattern of functionally associated genes such as cytokines, growth factors, laminins as well as keratins [8].

In the terminal stage, the kidneys of most patients with ESKD/ACKD have a largely similar morphological appearance regardless of the primary kidney disease. The inflammatory microenvironment in ESKD/ACKD, including the intense proliferation of fibroblasts, smooth muscle and endothelial cells and the remodelling of the extracellular matrix (ECM), bears characteristics of prolonged wound healing and resembles the tumour microenvironment [9]. A stepwise analysis of disease progression from initial cellular damage and inflammation towards fibrotic remodelling and loss of kidney function is not possible because the kidney is not removed, mainly due to clinically detectable tumour, until the final stage of disease progression. The aim of this study was to review the possible steps of disease progression from the reversible acute inflammatory phase towards cell proliferation, ECM alteration and renal remodelling to tumour development. We also considered the results of our previous gene expression study [8], as well as studies on inflammation, wound healing, and tumour development. We localised the selected genes to histological lesions, including tumour precursor lesions, and tumours in ESKD/ACKD by immunohistochemistry.

## Materials and methods

### Tissue samples

Twelve kidneys (6 ESKD and 6 ACKD) removed by radical nephrectomy due to cancer were collected from several European countries between 1995–1998 (see Acknowledgements). Kidneys were cut into 3-mm-thick slices and worked up entirely in 43–137 paraffin blocks for histological analysis. The H&E-stained slides were scored for cysts, microscopic precursor lesions and tumours, as previously described [10]. Cysts were classified as simple cysts lined with a single layer of epithelial cells and proliferative cysts lined with a tubulo-papillary growth of hyperplastic epithelial cells. The tumour was diagnosed according to the Heidelberg Classification [11] and as proposed by Tickoo et al. [6]. Among the 12 kidneys, 20 macroscopically detectable tumours were observed, including five papillary renal cell carcinomas (pRCCs), 6 conventional RCCs (cRCCs), one renal oncocytoma (RO), three ACKD-associated eosinophilic vacuolated RCCs (evRCCs), four chromophobe-like RCCs (chlRCCs), and one clear cell pRCC. We obtained three evRCCs from other institutes for consultation. The size of the main tumours varied between 30- and 40-mm. The evRCCs displayed papillary or solid growing large epithelial cells with eosinophilic cytoplasma containing vacuoles of variable size [6]. In addition to the main tumours, 65 small papillary, 40 chromophobe-like and 24 eosinophilic vacuolated pre-neoplastic lesions were noted. Three of the six cRCCs were accompanied by 3 or 4 small clear cell precursor lesions.

### Immunohistochemistry

Four µm paraffin sections of the affected kidneys were used for immunohistochemistry. Two slides from each selected kidney were dewaxed in xylene and rehydrated in graded ethanol. Antigen unmasking was performed by boiling the slides in 10 mM sodium citrate buffer, pH 6.0 or EnVision FLEX Target Retrieval Solution, high pH (DAKO, Glostrup, Denmark) in a 2100-Retriever (Pick-Cell Laboratories, Amsterdam, The Netherlands). Endogenous peroxidase activity and non-specific staining were blocked with Envision FLEX Peroxydase Blocking Reagent (DAKO) for 10 min at room temperature. Slides were then incubated for one hour in moist chamber with the antibodies listed in Table 1. EnVision FLEX horseradish peroxidase conjugated secondary antibody (DAKO) was applied for 30 min at room temperature. The signal was visualised with DAB (3,3’-Diaminobenzidin) or AEC (3-amino-9-ethylcarbazole) (DAKO). Tissue sections were counterstained with Mayer's haematoxylin (Lillie’s modification, DAKO) and after 10 s bluing in ammonium-hydroxide solution were mounted by Glycergel (DAKO). Photographs were taken with a Leitz DMRBE microscope, equipped with HC PLAN APO 20 × 0.70 objective, and a ProgRes C14 camera.

**Table 1:** Primary antibodies used for immunohistochemistry

Antibody	Reference	Species	Clonality	Dilution	Source
IL6	Anti-IL6 PA1-26,811	Rabbit	Poly	1:200	Thermo Fisher
CSF2	Anti-CSF2 TA808009	Mouse	Mono	1:150	Origene
TGFβ	Anti- TGFβ SAB4502954	Rabbit	Poly	1:100	Sigma-Aldrich
IL1β	Anti-IL1β AM06692SU-N	Mouse	Mono	1:600	Origene
αSMA,	Anti-αSMA ab124964	Rabbit	Mono	1:1000	Abcam
FN1	Anti-FN1 ab 32419	Rabbit	Mono	1:250	Abcam
LAMA3	Anti-LAMA3 HPA009309	Rabbit	Poly	1:50	Sigma-Aldrich
LAMB3	Anti-LAMB3 HPA008069	Rabbit	Poly	1:200	Sigma Aldrich
LAMC2	Anti-LAMC2 HPA024638	Rabbit	Poly	1.1000	Sigma-Aldrich
MMP9	Anti-MMP9 AV33090	Rabbit	Poly	1:200	Sigma-Aldrich
MMP12	Anti-MMP12 NBP1-31225	Rabbit	Poly	1:400	Novus Biol

## Results

**Fig. 1:**
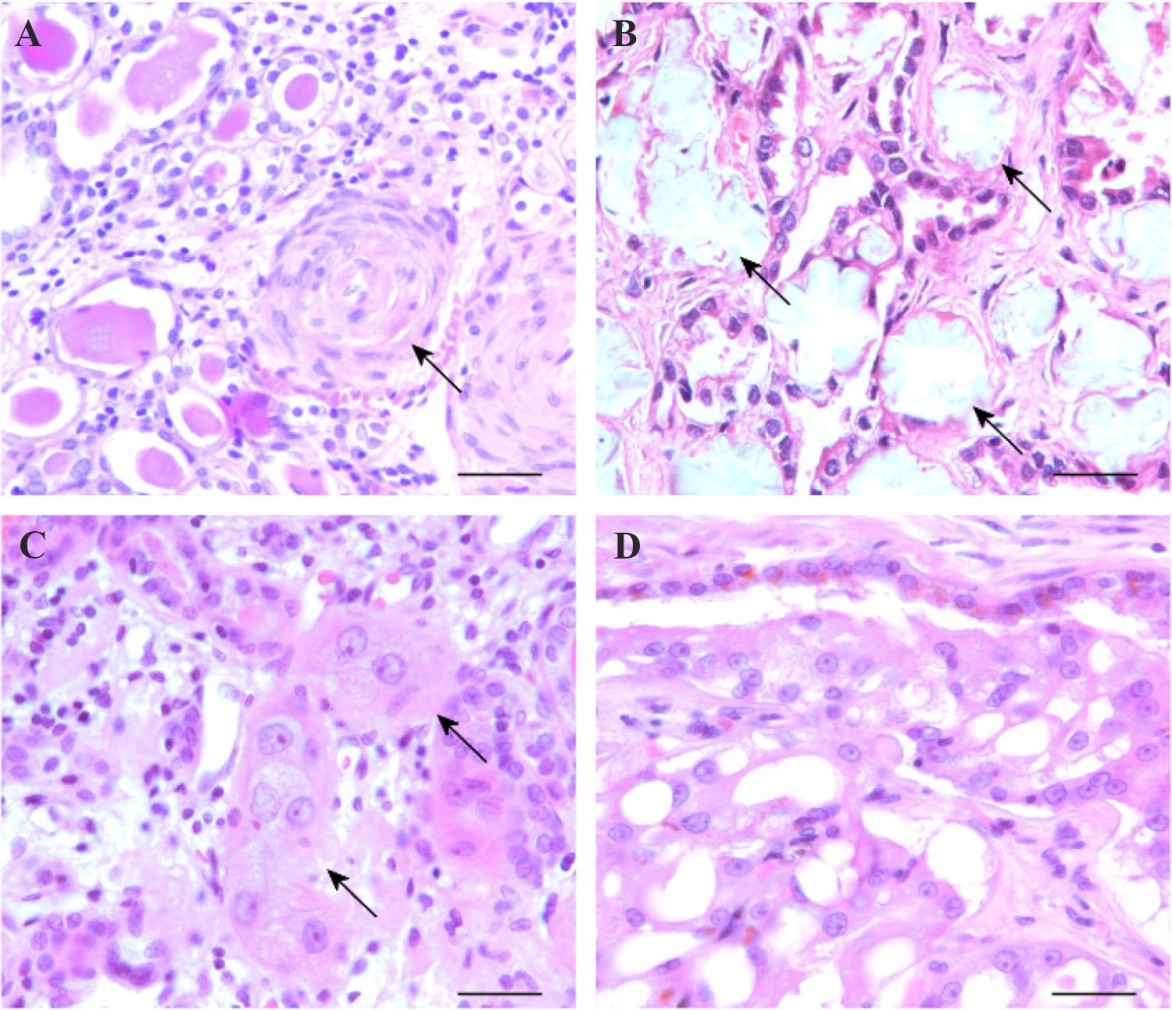
Characteristic histology of ESKD. **A** strong proliferation of myofibroblasts and myoendothelial cells around and within a small artery embedded in the inflammatory stroma of the ESKD sample(arrow). **B** ESKD-characteristic oxalate crystal deposition in fibrotic stroma with proliferation of epithelial cells (arrows). **C** Large epithelial cells showing enlarged nuclei and prominent nucleoli embedded in the inflammatory stroma of ESKD samples (arrows). **D** Eosinophil vacuolated cells growing within a cyst in ACKD samples. Scale bar: 35 μm

The histological characteristics of the kidneys (6 ESKD and 6 ACKD) analysed in this study were confirmed by evaluation of all slides. In H&E-stained slides of the ESKD samples, atrophic tubules, strong proliferation of endothelial and smooth muscle cells in arteries or arterioles, and an abundance of inflammatory fibrotic stroma were seen (Fig. 1A). Acquired cystic renal disease samples showed intensive cystic changes, but solid areas displayed stromal fibrosis and inflammation as in ESKD samples. Focal deposits of calcium oxalate crystals were detected in four cases (Fig. 1B). Isolated single epithelial cells or small epithelial cell clusters without lumen and visible basement membrane were seen in several slides (Fig. 1C). In the ACKD samples, cysts were lined with medium-sized cuboidal cells forming papillary structures. Some of the cysts were filled with large eosinophilic-vacuolated cells growing in a papillary-solid pattern, presumably precursors of evRCC (Fig. 1D). The tumours diagnosed as cRCC or pRCC showed a histology similar to that found in such tumours in the general population. The ACKD-associated evRCC displayed large vacuolated cells arranged in a solid-tubular pattern (Fig. 1D).

**Fig. 2:**
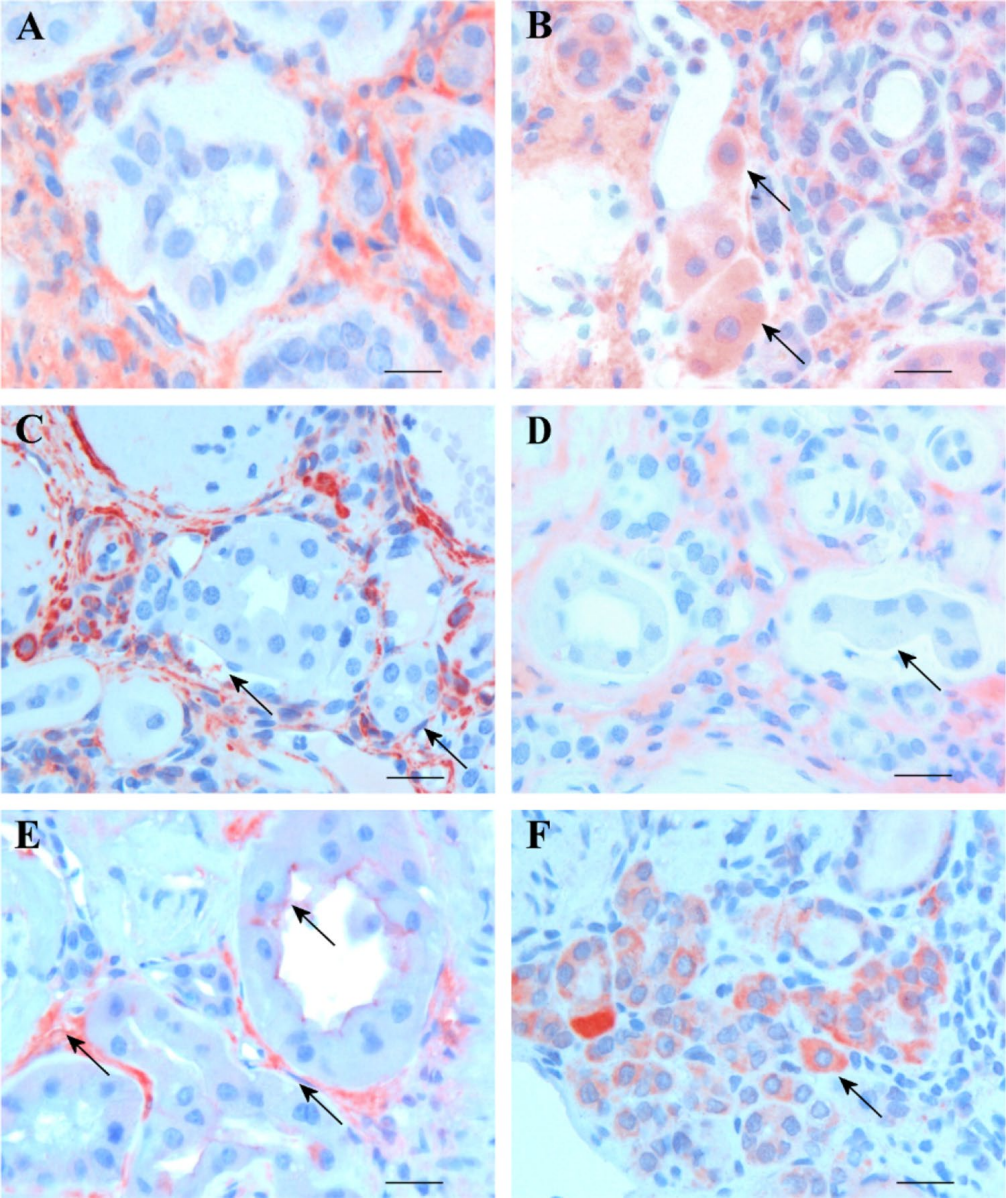
Cellular localisation of genes expressed in ESKD/ACKD samples. **A** Strong IL6 expression in stromal cells surrounding tubules and single epithelial cells. Note the collagen deposition around the tubule. **B** IL6 expression in stromal fibroblasts and in the cytoplasm of large proliferating epithelial cells (arrows). **C** Activated myofibroblasts showing aSMA expression around cell groups or single cells without visible basal membrane (arrows). **D** Fibronectin expression in the fibrotic-inflammatory stroma in ESKD samples. Note the small epithelial cell cluster surrounded by collagen (arrow). **E** MMP9 is expressed in stromal cells as well as at the basal and luminal surface of proliferating tubular cells (arrows). **F** Expression of MMP12 in tubules and single epithelial cells (arrow). Scale bar: 35 μm

Immunohistochemistry was employed to localise the genes listed in Table 1 in cellular components of ESKD/ACKD. Immunohistochemistry revealed strong IL6 expression in proliferating naïve activated fibroblasts (Fig. 2A). Small groups of epithelial cells without basal membrane (as shown in Fig. 1C) displayed positive IL6 immunreaction (Fig. 2B). Weak to strong IL6 immunostaining occurred in papillary epithelial cells growing within cysts in ACKD. TGFβ expression was detected in small epithelial cell clusters, proliferating pericytes and myofibroblasts around small arteries. We also found a strong expression of TGFβ in small cuboidal cells as well as large proliferating eosinophil-vacuolated cells lining smaller or larger cysts in the ACKD samples. The strongest expression of CSF2 was seen in proliferating endothelial cells and smooth muscle cells of arteries or arterioles, a weak expression of CSF2 was seen in irregularly growing tubular epithelial cells and single or clustered epithelial-like cells. No IL1β expression was seen in epithelial or stromal cells.

Strong αSMA expression was detected exclusively in naïve activated fibroblasts, none of the epithelial cells displayed positive staining. Proliferating myofibroblasts around small arteries and activated myoendothelial cells, substantially narrowing the arterial lumen, were positive for αSMA. In sclerotic inflammatory microenvironment, αSMA positive naïve activated fibroblasts and inflammatory cells dominated. Proliferating tubular cells, some without visible basal membrane, and smaller or larger cysts were surrounded by αSMA positive inflammatory fibroblastic stroma (Fig. 2C).

A positive immunostain with the antibody against FN1 was seen in the sclerosing stroma of ESKD/ACKD samples (Fig. 2D) and in myofibroblasts around small arteries. The FN1 immunohistochemistry showed the same staining pattern in all ESKD/ACKD samples as seen with the IL6 and αSMA antibodies. Occasionally, single epithelial cells displayed weak FN1 positivity. Antibodies to three components of laminin332, LAMA3, LAMB3 and LAMC2 showed positive immune reaction in ESKD/ACKD samples. LAMA3 and LAMB3 were expressed in groups of small epithelial-like cells and in the fibrotic stroma, whereas expression of LAMC2 occurred preferentially in epithelial cells.

The expression of MMP9 was detected in stromal fibroblasts surrounding tubules and small epithelial cell clusters in ESKD/ACKD (Fig. 2E). In ACKD, some of the epithelial cells lining larger cysts also expressed MMP9, and strong expression of MMP9 was seen in naïve activated fibroblasts around the cysts. Immunohistochemical examination of MMP12 revealed cytoplasmic staining in epithelial cells of dilated tubules, smaller or larger cysts of ACKD samples and in small, aggregated tubules (Fig. 2F). Moreover, weak to strong MMP12 staining intensity was observed in single epithelial-like cells and cell groups separated by fibrotic stroma. No MMP12expression was detected in stromal fibroblasts.

## Discussion

The development of ESKD parallels the wound healing paradigm of inflammation, proliferation, and remodelling [9]. Proximal tubular cells play a crucial role in triggering inflammation. Reduced oxygen and nutrient delivery or toxic stresses lead to the damage of proximal tubules. Under normal circumstances, the proximal tubular system can fully or near fully recover from a short-lasting stress [12]. However, the situation changes dramatically during prolonged ischaemic, toxic, or metabolic stress, when the damaged proximal tubular cells cannot recover and continuously express proinflammatory cytokines such as IL6, CSF2 and TNFα [13]. The production of cytokines by resident or recruited immune cells increases the initial inflammatory signal into a positive loop of amplification. The final outcome of this inflammatory loop is the proliferation of naïve activated fibroblasts, leading to overproduction of ECM components and remodelling of the kidney structure with subsequent loss of tissue integrity and renal function.

IL-6 seems to be the main driver of progressive inflammation and is expressed by naïve activated fibroblasts and by proliferating epithelial cells [10]. IL-6 enhances the synthesis of the acute phase proteins lipopolysaccharide binding protein and serum amyloid A1 by stromal cells [14]. The lipopolysaccharide binding protein catalyses signalling to a receptor complex including toll like receptor 4 via a CD14-enhanced mechanism, resulting in the release of pro-inflammatory cytokines such as IL-6, which in turn enhances lipopolysaccharide binding protein synthesis [15]. Stimulation of toll like receptor 4 by the lipopolysaccharide binding protein-CD14 complex promotes the NFkB-dependent up regulation of pro-inflammatory cytokines and matrix metalloproteinases via MyD88 activation, all of which are important for remodelling processes [16, 17].

The inflammatory microenvironment is composed of distinct cellular (fibroblasts, endothelial and inflammatory cells) and non-cellular (cytokines, proteoglycans, collagens, fibronectins, and proteases) components. In this study, we showed the presence αSMA-expressing naïve activated fibroblasts, proliferation of endothelial cells and expression of inflammatory cytokines. Fibrillar elements such as FN1, laminin332 and ECM modifier enzymes were abundantly detected in ESKD/ACKD samples. Our present data strongly suggest the role of a continuous “wound healing programme” in kidney remodelling. Naïve activated fibroblasts play a crucial role in wound healing but are only transiently activated until the end of the healing process [18]. However, in ESKD/ACKD, naïve activated fibroblasts remain perpetually activated due to continuous cellular damage, inflammation, and increased level of reactive oxygen species. Naïve activated fibroblasts communicate with epithelial cells via inflammatory cytokines such as IL-6, TGFβ and fibroblast growth factor, like cancer-associated fibroblasts with cancer cells [19]. Naïve activated fibroblasts promote the remodelling of ECM by producing MMPs [17].

Fibronectin is synthesised by naïve activated fibroblasts, activated smooth muscle and endothelial cells, all of which are abundant in ESKD/ACKD. Fibronectin is involved in cell adhesion, cell proliferation and migration processes during embryogenesis and is re-expressed during wound healing and blood coagulation. Fibronectin is essential in initiating the assembly of ECM proteins such as collagen, fibrillin, fibrinogen, fibulin, integrins and thrombospondin and plays therefore a role in remodelling the ECM in wound healing [20]. Through interaction with other ECM components, laminin332 mediates cell attachment and migration, and the organsation of cell structures during embryonic development, regeneration, tissue remodelling, tumourigenesis and metastasis [21].

Matrix modifier proteases play a fundamental role in ECM degradation in normal physiological processes during embryonic development, wound healing, tissue remodelling and cancer spread [17]. In ESKD/ACKD, MMP9 expressed by naïve activated fibroblasts and macrophages contributes to basement membrane degradation and ECM protein network remodelling. Naïve activated fibroblasts can trigger the production of matrix modifier proteases with ECM remodelling capacities and can also promote epithelial to mesenchymal transition and regulate the migration and dissemination not only of tumour cells but also normal epithelial cells [22]. Delaminated epithelial cells, especially small epithelial cell groups or single cells seen in ESKD/ACKD, may migrate into the inflammatory stroma and may be a precursor lesion for tumour development. Migration of displaced epithelial cells is an important step in tumourigenesis and cancer progression [22]. Recently, MMP12 expression has been shown to be significantly associated with cRCC progression, confirming its involvement in tumour cell migration [23].

The strong inflammatory response during kidney remodelling in ESKD/ACKD induces the production of high levels of reactive oxygen species by macrophages and naïve activated fibroblasts embedded in the ECM. One of the common mediators of carcinogenesis is the imbalance of oxidative stress induced by inflammation. Expression of IL6 and TGFβ in ESKD/ACKD triggers the generation of reactive oxygen species in both phagocytic and non-phagocytic cells in the ECM. Reactive oxygen species cause mitochondrial and genomic DNA damage, which may also contribute to tumour development [24, 25]. During remodelling, the epithelial cells of the nephron gradually lose their function, suspend their terminal differentiation programme and shift their keratin expression profile from KRT8 and KRT18 to KRT7 and KRT19, allowing them to change shape and increase plasticity, both of which are necessary for remodelling and tumourigenesis [26].

**Fig. 3:**
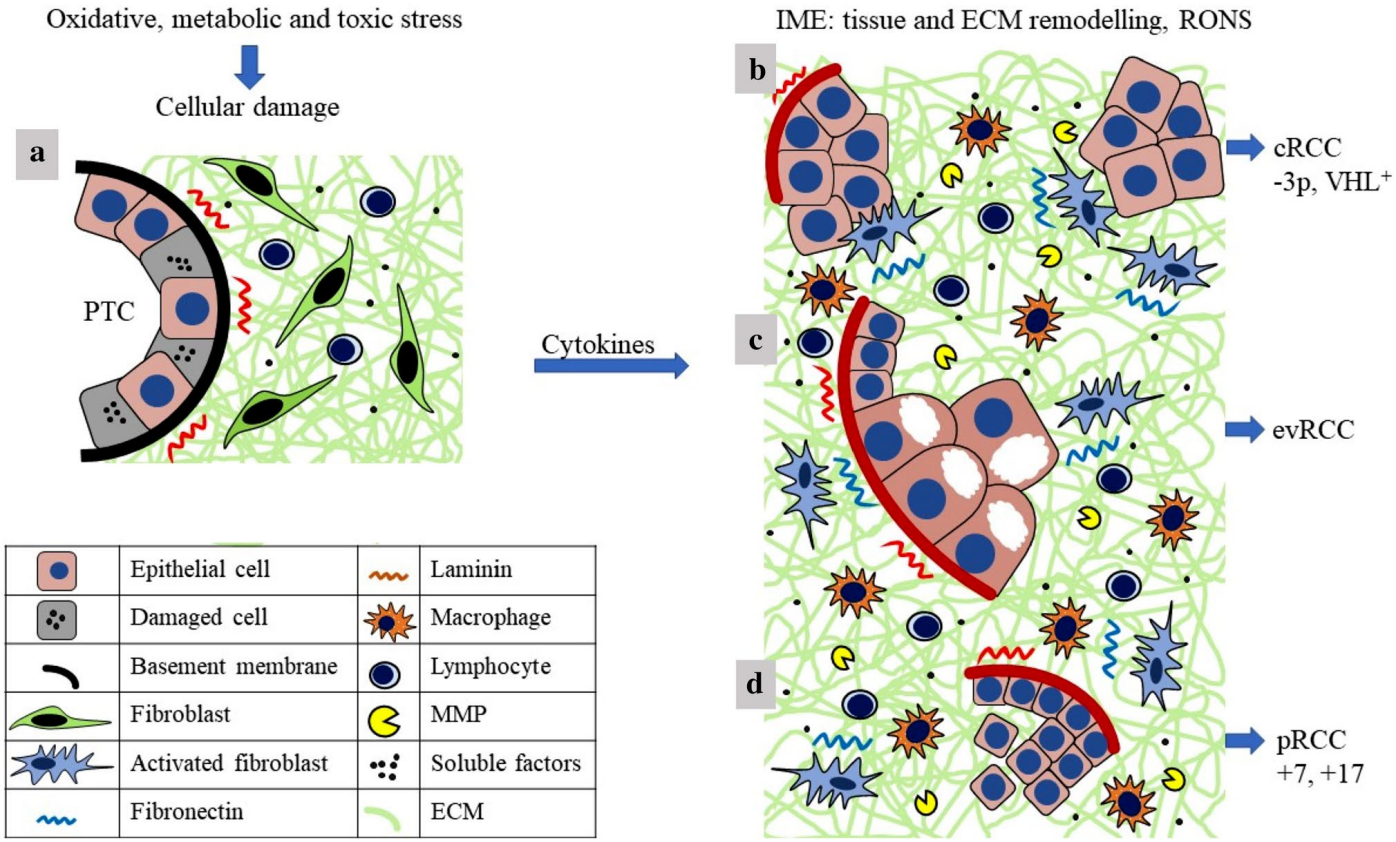
Proposed pathways of renal tumour development in ESKD/ACKD. See discussion for explanation

Histological, immunhistochemical and molecular studies on ESKD/ACKD recall Virchow’s observation that cancers preferentially occur at sites of chronic inflammation [27]. There is well-documented association between chronic inflammation and cancer risk, such as viral hepatitis and hepatocellular carcinoma, Helicobacter pylori infection and gastric cancer, Schistosomiasis and squamous cell bladder cancer, or human papilloma virus and cervical cancer [28]. The association between chronic inflammation and tumour development in ESKD/ACKD can be added to the above examples. There are distinct types of renal cancer in the general population [11]. Given our knowledge of the development and natural history of RCCs in the general population, we propose developmental sequences of cRCC, pRCC and evRCC occurring in ESKD/ACKD (Fig. 3).

Conventional RCC originates from proximal tubular cells and is characterised by a deletion of chromosome 3p and a mutation of the VHL and PRMB1 genes (Fig. 3b). The loss of chromosome 3p in the karyotype of normal adult kidney cells is not a rare alteration. The high reactive oxygen species concentration in the inflammatory microenvironment may cause mutation of the VHL and/or PRMB1 genes, both of which are located on chromosome 3p. The loss of chromosome 3p and VHL and/or PRMB1 mutation in the same epithelial cell may change its behaviour in favour of tumour development. TGFb1-mediated activation and migration of tubular cells as well as increased production of matrix modifier proteases that dissolve the basement membrane play an important role in cRCC development. The role of tumourigenic inflammatory microenvironment is supported by the observation that cRCC in the general population occurs as a solitary tumour without precursor lesions, whereas in ESKD/ACKD it is accompanied by several small precursor lesions. Almost all cRCCs arising in ESKD/ACKD and in the general population share common genetic alterations, suggesting a shared natural history.

The so-called ACKD-associated evRCC occurs exclusively in ACKD. In a large multi-institutional study on ACKD, nearly every second tumour was diagnosed as evRCC [6]. The occurrence of evRCC is associated with multiple pre-neoplastic lesions that have a similar histological pattern [10]. We observed several cysts lined by medium-sized cuboidal cells and filled with large eosinophilic vacuolated tumour cells. The origin of large eosinophilic-vacuolated cells is unknown; they might derive from cystic-papillary precursor lesions (Fig. 3c). Since evRCC occurs exclusively in ACKD which develops primarily in patients on longterm dialysis, it is reasonable to assume that the unique morphology of evRCC is related to dialysis. No consistent genetic alterations have been detected in evRCCs.

There is debate about the origin of papillary RCCs in the general population and in ESKD/ACKD. It has been suggested that papillary RCC arises from not fully differentiated embryonic rests persisting during life [29]. The high frequency of small papillary lesions in the general population was documented by Apitz in 1944 [30]. He suggested that these lesions develop due to disturbance of cellular differentiation in the embryonic kidney, and renal sclerosis only increases the number and size of these lesions. Similarly, pRCC in ESKD/ACKD may develop from persisting embryonic rests. The overwhelming majority of lesions in ESKD/ACKD display a papillary growth pattern [7]. Papillary lesions and tumours arising in ESKD/ACKD and in the general population share common genetic alterations such as trisomy 7 and 17. These data strongly indicate that papillary lesions detected in ESKD/ACKD develop from embryonic rest cells and start to proliferate in a protumourigenic inflammatory microenvironment, as suggested by Apitz [30].

In conclusion, we suggest that long lasting inflammation, remodelling of the kidney structure and the change of ECM from anti-tumourigenic to protumourigenic may be responsible for the high frequency of carcinomas, especially of evRCC, in ESKD/ACKD. A few years ago, it was suggested that cancer is a never healing wound [18]. Similarly, we can refer to the progressive inflammatory and fibrotic processes in ESKD/ACKD as a never healing wound, which may ultimately lead to another never healing wound, renal cancer [31].

A limitation of our study is that no data are available on the patients' inflammatory parameters. In addition, the diagnosis of the original disease leading to ESKD/ACKD and the duration of dialysis are not available.

## Data Availability

All histological slides and representative slides of immunohistochemistry are available from the corresponding author on reasonable request.

## References

[1] US. Renal Data System (2013) USRDS 2013. Annual Data Report: Atlas of End-Stage Renal Disease in the United States, National Institutes of Health, National Institute of Diabetes and Digestive and Kidney Diseases, Bethesda, MD

[2] Hughson MD, Buchwald D, Fox M (1986). Renal neoplasia and acquired cystic disease in patients receiving long-term dialysis. Arch Pathol Lab Med.

[3] Dunnill MS, Millard PR, Oliver D (1997). Acquired cystic disease of the kidneys: a hazard of long-term intermittent maintenance haemodialysis. J Clin Pathol.

[4] Matson MA, Cohen EP (1990). Acquired cystic kidney disease: occurrence, prevalence, and renal cancers. Medicine (Baltimore).

[5] Chudek J, Herbers J, Wilhelm M, Wilhelm M, Kenck C, Bugert P (1998). The genetics and morphology of renal cell tumors in end-stage renal failure may differ from those occurring in the general population. J Am Soc Nephrol.

[6] dePeralta-Venturina TSK, Harik MN, Worcester LR, Salama HD, Young ME (2006). Spectrum of epithelial neoplasms in end-stage renal disease: an experience from 66 tumor-bearing kidneys with emphasis on histologic patterns distinct from those in sporadic adult renal neoplasia. Am J Surg Patho.

[7] Kovacs G (1995). High frequency of papillary renal cell tumors in end stage kidneys-is there a molecular genetic explanation? Editorial Comment. Nephrol Dial Transplant.

[8] Nagy A, Walter E, Zubakov D, Kovacs G (2016). High risk of development of renal cell tumor in end stage kidney disease: the role of microenvironment. Tumor Biol.

[9] Gál P, Varinska L, Fáber L, Novak S, Szabo P, Mitrengova P (2017). How signaling molecules regulate tumor microenvironment: parallels to wound repair. Molecules.

[10] Peterfi L, Yusenko MV, Kovacs G (2019). IL6 shapes an inflammatory microenvironment and triggers the development of unique types of cancer in end-stage kidney. Anticancer Res.

[11] Kovacs G, Akhtar M, Beckwith BJ, Bugert P, Cooper CS, Delahunt B (1997). The Heidelberg classification of renal cell tumours. J Pathol.

[12] Lindgren D, Boström AK, Nilsson K, Hansson J, Sjölund J, Möller C (2011). Isolation and characterisation of progenitor-like cells from human renal proximal tubules. Am J Pathol.

[13] Daha MR, van Kooten C (2000). Is the proximal tubular cell a proinflammatory cell?. Nephrol Dial Transplant.

[14] Schumann RR, Kirschning CJ, Unbehaun A, Aberle HP, Knope HP, Lamping N (1996). The lipopolysaccharide binding protein is a secretory class 1 acute-phase protein whose gene is transcriptionally activated by APRF/STAT/3 and other cytokine-inducible nuclear proteins. Mol Cell Biol.

[15] Faure E, Equils O, Sieling PA (2000). Bacterial lipopolysacchride activates NF-kappaB through toll-like receptor 4 (TLR-4) in cultured human dermal endothelial cells. Differential expression of TLR-4 and TLR-2 in endothelial cells. J Biol Chem.

[16] Gluba A, Banach M, Hannam S, Mikhailidis DP, Sakowicz A, Rysz J (2010). The role of Toll-like receptors in renal diseases. Nat Rev Nephrol.

[17] Kessenbrock K, Plaks V, Werb Z (2010). Matrix metalloproteinases: regulators of the tumor microenvironment. Cell.

[18] Dvorak HF (1986). Tumors: wounds that do not heal. similarities between tumor stroma generation and wound healing. N Eng J Med.

[19] Cirri P, Chiarugi P (2011). Cancer associated fibroblasts: the dark side of the coin. Am J Cancer Res.

[20] Tracy LE, Minasian RA, Caterson EJ (2016). Extracellular matrix and dermal fibroblast function in the healing wound. Adv Wound Care.

[21] Marinkovich MP (2007). Tumour microenvironment: laminin 332 in squamous-cell carcinoma. Nat Rev Cancer.

[22] Nguyen-Ngoc KV, Cheung KJ, Brenot A, Shamir ER, Gray RS, Hines WC (2012). ECM microenvironment regulates collective migration and local dissemination in normal and malignant mammary epithelium. Proc Nat Acad Sci USA.

[23] Beres B, Yusenko M, Peterfi L, Kovacs G, Banyai D (2022). Matrix metalloproteinase 12 is an independent prognostic factor predicting postoperative relapse of conventional renal cell carcinoma - a short report. Cell Oncol (Dordr).

[24] Nagy A, Wilhelm M, Kovacs G (2003). Mutations of mtDNA in renal cell tumours arising in end-stage renal disease. J Pathol.

[25] Bertram C, Hass R (2008). Cellular responses to reactive oxygen species-induced DNA damage and aging. Biol Chem.

[26] Sarlos DP, Peterfi L, Szanto A, Kovacs G (2018). Shift of keratin expression profile in end stage kidney increases the risk of tumor development. Anticancer Res.

[27] Virchow R (1863). Die krankhaften Geschwülste (Dreissig Vorlesungen, gehalten wahrend des Wintersemesters 1862–1863).

[28] Balkwill F, Charles KA, Mantovani A (2005). Smoldering and polarized inflammation in the initiation and promotion of malignant disease. Cancer Cell.

[29] Banyai D, Sarlos DP, Nagy A, Kovacs G (2018). Recalling Cohnheim's theory: Papillary renal cell tumor as a model of tumorigenesis from impaired embryonal development to malignant tumors in adults. Int J Biol Sci.

[30] Apitz K (1944). Die Geschwülste und Gewebsmissbildungen der Nierenrinde. Die Adenoma Virchows Arch.

[31] Rybinski B, Franco-Barraza J, Cukierman E (2014). The wound healing, chronic fibrosis, and cancer progression triad. Physiol Genom.

